# Tooth Wear Prevalence and Associated Risk Factors Among a Small Portuguese Community: A Retrospective Study

**DOI:** 10.3390/jcm14061810

**Published:** 2025-03-07

**Authors:** Rui Carvalho, Sofia Rodrigues, Manuel Nobre, João Rua, Eduardo Guerreiro, Luís Proença, Ana M. Vieira

**Affiliations:** 1 Clinical Research Unit (CRU), Egas Moniz Center for Interdisciplinary Research (CiiEM), Egas Moniz School of Health & Science, 2829-511 Almada, Portugal; mnobre@egasmoniz.edu.pt (M.N.); jrua@egasmoniz.edu.pt (J.R.); eguerreiro@egasmoniz.edu.pt (E.G.); lproenca@egasmoniz.edu.pt (L.P.); asvieira@egasmoniz.edu.pt (A.M.V.); 2 Egas Moniz University Clinic, Egas Moniz School of Health & Science, 2829-511 Almada, Portugal

**Keywords:** tooth wear, grading index scales, risk factors, prevalence

## Abstract

**Background/Objectives**: Early tooth wear (TW) assessment prevents challenging and costly dental treatments. Knowledge of data on the prevalence and risk factors is crucial for developing preventive guidelines. This retrospective cross-sectional study aims to study the prevalence of TW in a sample of patients seeking a Screening and Emergency appointment at Egas Moniz Dental Clinic (EMDC) and its associated risk factors. **Methods**: Descriptive and logistic regression analysis were applied to data from a sample of 2266 patients, collected between 2021 and 2023, to ascertain the presence of tooth wear, its different types and its correlation with potential risk factors. **Results**: The prevalence of dental wear was found to be 54.7%, with attrition (24.1%) being the most prevalent lesion and erosion (2.7%) the least prevalent. Concerning the risk predictors for tooth wear, age (OR = 1.01–1.05, *p* < 0.05), decreased DVO (OR = 2.16, *p* = 0.028), antacids (OR = 7.07, *p* = 0.003), mastication difficulties (OR = 1.87, *p* = 0.039), drugs (OR = 2.38, *p* = 0.032) and use of mouthwash (OR = 1.47, *p* = 0.008) were positively associated. Gender (OR = 0.7, *p* = 0.015) was negatively associated, with females showing a 30% less risk than males. **Conclusions**: It was concluded that TW is prevalent and increases with age, underscoring the necessity for timely and accurate diagnosis to minimize its progression. Additionally, it is crucial to conduct a thorough evaluation of the risk factors involved to implement effective preventive measures and treatment strategies.

## 1. Introduction

Tooth wear (TW) is a multifactorial condition described as the progressive loss of dental hard tissues [1,2]. Due to its multifactorial etiology, TW can manifest in many ways and can be difficult to diagnose and to manage [2]. It occurs mainly through three specific processes: abrasion, attrition and erosion. A fourth wear-related process, abfraction, was postulated to potentiate wear by abrasion and/or erosion, although this term has been recently discouraged as the current level of evidence to justify it as a separate process is considered to be weak [1,3]; also, the term “non-carious cervical lesion” (NCCL) is a more appropriate designation to describe abrasion lesions [4].

It is important to prevent or reduce the destruction resulting from tooth wear by the early identification of TW lesions, to grade its severity, to diagnose the likely cause or causes and to monitor this condition [5]. The most recent consensus statement on the management of severe tooth wear states that priority should be given to the early detection of (severe) tooth wear and to identifying the underlying etiology [6,7].

Patients experiencing tooth wear face clinical challenges. Clinical implications may arise, as functional difficulties (chewing difficulties and loss of DVO), esthetic concerns, progressive damage and the need for more complex treatment approaches may lead to a restorative death spiral [6]. Also, the rehabilitation of such cases does not eliminate/prevent the wear, but rather modifies its rate, location and the underlying nature of wear, and can be more prone to failure when evaluating severe tooth wear patients due to bruxism or erosion and bruxism [6,8].

The global prevalence of tooth wear was evaluated in recent multi-center studies throughout Europe, Arabia and China, reporting a range from 29% to 60% [9,10,11]. The variation in data reflects the different methods used to quantify TW and the difference in ages [12]. Also, TW is becoming a relevant oral health issue among young adults, as cross-sectional studies have revealed that it occurs at a young age [13,14]. To date, in the Portuguese community, very few data have provided a comprehensive assessment of TW prevalence. Addressing the risk factors, only two studies were found regarding dental erosion in patients with alcoholic habits and vomiting behavior (anorexia nervosa and bulimia nervosa) [15,16].

This study aims at investigating the prevalence and related risk factors concerning TW in a population attending a Portuguese university dental clinic, contributing to solving the lack of data on this condition in this community, and to the development of effective strategies for its management and prevention. Our hypothesis is that patients with a higher consumption of acidic diets, frequent endogenous acid exposure or inadequate oral health or oral hygiene habits will exhibit a higher prevalence of TW.

## 2. Materials and Methods

### 2.1. Study Design, Eligibility Criteria and Sampling

This retrospective cross-sectional study was conducted at Egas Moniz Dental Clinic (EMDC) at Egas Moniz School of Health and Science University (Almada, Portugal). This was an uninterrupted data analysis study following a non-probability sampling technique from April 2021 to November 2023 and was approved by the Egas Moniz Ethics Committee (1349/2021). First-incoming patients seeking treatment through a Screening and Emergency appointment at EMDC were invited to voluntarily and anonymously participate in this study. To be included, patients had to be able to understand and sign the informed consent form, be 18 years of age or older and to have a completely filled tooth wear index at the appointment.

### 2.2. Data Collection

The first-incoming patients underwent a mandatory triage that included a self-reported health questionnaire, full-mouth clinical observation and radiographies. The self-reported questionnaire included age, sex, educational level, employment status, general medical history and medication, smoking habits and oral hygiene habits.

The patients were positioned in a dental chair under the examination light source, and the observations took place using a dental mirror, air syringe and cotton rolls. The data extracted for this particular study consisted of general patient information, such as age and sex, medication, oral hygiene habits, alcohol and drug use, diet and clinical observations. The examinations were conducted by 4th and 5th year dental students, supervised by qualified clinical assistants (R.P.C; M.N; E.G), who validated the final diagnosis.

### 2.3. Variables

The presence or absence of erosion, attrition and NCCL and the combinations of different wear types (erosion and/or attrition and/or NCCL) were the clinical outcome variables, registered as dichotomous variables (yes or no). The independent variables, or predictor variables, were sociodemographic and behavioral information, which were collected from the self-reported questionnaire. These were age (recorded as a continuous variable in years), gender (divided into two groups, male and female), dry mouth (never, occasionally, frequently, always), consumption of alcohol (during meals, socially, both), drug use, consumption of beverages (1 time a day, 2–3 times daily, more than 3 times daily, 1–3 times weekly, more than 3 times weekly, never), consumption of fresh fruits (1 time a day, 2–3 times daily, more than 3 times daily, 1–3 times weekly, more than 3 times weekly, never) and tooth brushing frequency (1 time a day, 2–3 times daily, more than 3 times daily, 1–3 times weekly, more than 3 times weekly, never). The following variables were registered as dichotomous variables (yes or no): mouthwash use, esthetic complaints, bronchodilators, antacids and mastication difficulties.

### 2.4. Statistical Analysis

Data analysis was performed using IBM SPSS Statistics version 29.0 (IBM Corp., Armonk, NY, USA). Descriptive and inferential statistics methodologies were applied.

Further, logistic regression analysis was used to model the relationship between TW and several risk indicators. Multivariate models were constructed for each of the outcome clinical variables (erosion, attrition and presence of NCCL) via a stepwise procedure. Only predictor variables showing a significance of *p* ≤ 0.05 in the univariate model were included in the multivariate logistic regression procedure. The contribution of each variable to the model was evaluated by Wald statistics. Interactions were also analyzed for all tested variables. A final reduced model for each of the considered clinical outcomes was obtained, based on the significant predictor variable categories. The Odds Ratio (OR) along with the correspondent 95% Confidence Interval (95% CI) were calculated for both the univariate (crude OR) and multivariate analyses (adjusted OR). The level of statistical significance was set at 5%.

## 3. Results

### 3.1. Patients Characteritics

The research sample consisted of 2266 patients who attended a Screening and Emergency appointment at EMDC and were considered eligible for inclusion in this study (Table 1). A total of 936 patients were males with a mean age of 42.85 years (±19.7 years) and 1330 were females with a mean age of 42.52 (±19.6 years).

### 3.2. Tooth Wear Prevelance

Attrition was the most prevalent form of tooth wear (24.1%) (Table 1). The least prevalent lesion type was erosion (2.7%). In the combined lesions, attrition + NCCL (10.4%) was the most prevalent, and erosion + NCCL the least prevalent (1.8%). A total of 1026 patients did not present any type of tooth wear (45.3%). Significant differences (*p* = 0.012) between males and females were only found for attrition + NCCL, with males showing a higher percentage (12.3%) of these combined lesions when compared to females (9%).

### 3.3. Risk Factors

Analysis of the risk factors as a function of gender (Table 2) showed significant differences for alcohol intake (*p* < 0.001) and narcotics (*p* < 0.001), with males showing higher values than females. Females showed significantly higher frequencies for fruit consumption (*p* = 0.026), mouthwash use (*p* = 0.021), mastication difficulties (*p* = 0.005), being ashamed to smile (*p* = 0.001) and frequent dry mouth (*p* = 0.007).

The predictor variables that showed significant associations in the multivariate analysis with the prevalence of different types of TW are presented in Table 3.

No predictor variables were associated with the presence of erosion. Age was positively associated with both attrition (OR = 1.01, *p* < 0.001) and NCCL (OR = 1.01, *p* = 0.004). A positive association with erosion + attrition was found for age (OR = 1.03, *p* < 0.001), antacids (OR = 7.07, *p* = 0.003) and mastication difficulties (OR = 1.87, *p* = 0.039). Erosion + NCCL was only positively associated with age (OR = 1.03, *p* < 0.001). Attrition + NCCL was positively associated with age (OR = 1.05, *p* < 0.001), drugs (OR = 2.38, *p* = 0.032) and mouthwash use (OR = 1.47, *p* = 0.008), and negatively associated with gender (OR = 0.70, *p* = 0.015), with females having 30% less risk of developing this type of combined wear than males. Erosion + attrition + NCCL was positively associated with age (OR = 1.05, *p* < 0.001) and decreased DVO (OR = 2.16, *p* = 0.028).

## 4. Discussion

The primary objective of this study was to evaluate the occurrence of TW among individuals attending a Screening and Emergency consultation at EMDC. Overall, the prevalence of TW was 54.7%, with attrition (24.1%) being the most prevalent type, similar to results seen in studies such as Al-Ani [17], Hedge et al. [18] and Hemmings et al. [19], followed by the NCCL (11.6%) and erosion (2.7%) types, considering individual lesions.

Dental hard tissue loss, resulting in TW, is a matter of growing significance; this is particularly true as people live longer and are able to maintain their natural teeth into old age [9,20,21]. TW can be caused by a range of factors, including chemical, biological and behavioral, as well as medications, gastrointestinal problems and acid regurgitation [9,20].

Documenting a comprehensive history for the primary complaint and finding the etiology is important in order to manage the associated risk factors, preventing further structural loss and allowing the monitoring of the associated symptoms [22]. Of the risk factors documented, mouthwash (42.1%) was a popular element included in the patient’s oral hygiene habits. Nonetheless, its abuse can cause dental erosion, one of the most reported side effects, not only because of the pH, but also due to its viscosity [23]. Factors such as decreased VDO (22.6%), mastication difficulties (19.3%) and frequent dry mouth (11.7%) also displayed moderate prevalence percentages. Additional to the primary objective, we aimed to explore any potential associations between TW and various risk factors.

Concerning risk predictors in individual TW lesions, none were positively associated with erosion. Age was a common predictor for attrition and NCCL (OR = 1.01 equally, *p* < 0.05). For erosion + attrition + NCCL, age (OR = 1.05, *p* < 0.001) and decreased DVO (OR = 2.16, *p* = 0.028) were significantly associated. For erosion + attrition, age (OR = 1.03, *p* < 0.001), antacids (OR = 7.07, *p* = 0.003) and mastication difficulties (OR = 1.87, *p* = 0.039) were significant. Considering erosion + NCCL, only age (OR = 1.03, *p* < 0.001) was positively associated. Regarding attrition + NCCL, age (OR = 1.05, *p* < 0.001), narcotics (OR = 2.38, *p* = 0.032), mouthwash (OR = 1.47, *p* = 0.008) and female gender (OR = 0.7, *p* = 0.015) were significantly associated.

In accordance with this investigation, age has demonstrated itself to be a common significant result when associated with TW, as seen in works such as Shrestha & Rajbhandari [24], Sun et al. [25], Van’t Spijker et al. [10] and Wei et al. [26]. Considering gender, Wei et al. [26] and Zhang et al. [27] concluded that there was no distinction in prevalence between genders. Nevertheless, our results indicated a higher prevalence of TW in the male gender (58.2%). Despite this, our multivariable analyses did not reveal a positive association between TW and gender, except for when considering the association between attrition and NCCL, where female gender revealed a lower risk (30%, OR = 0.7). Also, our study observed no correlation between tooth wear and how often individuals brushed their teeth, which is consistent with the findings of Sadaf et al. [28] and Bartlett et al. [29]. On the other hand, research by Wei et al. [30] demonstrated a significant correlation between these variables.

Among patients who frequently consumed acidic foods and soft drinks, this investigation found no increase in TW, which is in accordance with the findings of studies conducted on Chinese adults [25]. In contrast, research conducted in China, Poland and Europe demonstrated a significant association between the frequent consumption of these items and increased TW [26,29,30]. These differences must be interpreted with caution, since self-report questionnaires for diet intake may lead to underreporting [31], and are subject to measurement errors and biases [32]. Concerning the interaction between attrition + NCCL and drug use, there were substantial findings. This might be explained by the presence of bruxism as a result of drug-induced hyperactivity [33,34]. According to Donaldson & Goodchild [33] and Shetty et al. [34], erosion is also common in cases of substance abuse; however, this is not in line with our results.

Intrinsic acid from conditions such as GERD, anorexia and bulimia can also lead to erosive TW, as demonstrated by other studies [25,26,29,30]; however, this was not supported by the results found in the present study. Interestingly, the intake of antacids (OR = 7.07) was proven significant when correlated with the combined lesions of erosion and attrition, which leads us to think that some patients may exhibit signs of erosion due to other pathologies treated with such medication, rather than GERD, or that GERD was not correctly diagnosed.

In this study, the frequent sensation of dry mouth was not significantly linked to tooth wear caused by erosion. This finding differs from the results of Sun et al. [25] and Zwier et al. [35], who found that low salivary flow is a major risk factor for tooth wear. Both studies highlighted the vital role of saliva in maintaining oral health, particularly in protecting teeth from erosion by neutralizing acids and supporting enamel repair [25,35].

However, individuals with xerostomia may adopt compensatory measures, such as using saliva substitutes or maintaining strict oral hygiene, minimizing the impact of reduced saliva flow on tooth wear. Furthermore, the Mediterranean diet, common in Portugal (more than 26% in 2020), is characterized by less acidic foods, which may reduce the incidence of dental wear even in the presence of xerostomia by the close relation of this diet to reducing and limiting the acid action [36,37,38].

Also, the lack of correlation between the sensation of dry mouth and TW found in the present study might reflect the subjectivity of this risk factor, as xerostomia is usually (and in this particular study) self-reported, and a direct correlation between the sensation of dry mouth and actually reduced levels of saliva production cannot be determined, emphasizing the need for effectively measuring saliva parameters; for example, the evaluation of salivary flow (stimulated and not stimulated) [39].

This research is a retrospective study, which comes with certain limitations. For instance, there may be incomplete information about exposure to causal agents. Retrospective studies that depend on self-reported data are prone to biases, as participants may misremember past events, leading to the misclassification of exposures or outcomes, or may modify their responses to conform to societal expectations [40]. Therefore, it is crucial to acknowledge and account for potential biases in retrospective research, particularly when using subjective data collection methods such as self-reported habits. Another challenge is the difficulty in differentiating between erosion, attrition, and abrasion, as these conditions frequently coexist and overlap, making clear distinctions hard to establish, and since identifying early signs and symptoms of wear, particularly those which are erosion-related, has proven to be difficult [41,42].

Nevertheless, given the lack of clinical studies on TW and its associated risk factors in large populations, this study might give some insight into the planning of more controlled clinical trials on this topic and contribute to the development of prevention and monitoring strategies.

## 5. Conclusions

The prevalence of TW reported in this study was 54.7%, with attrition being the most common lesion (24.1%) and erosion the least frequent (2.7%).

Several risk factors were associated with TW, in both individual and combined lesions, with age present in most wear type combinations as a statistically significant risk factor.

Further studies should be conducted to delve deeper into the reasons behind the findings and to formulate less ambiguous questions for a better understanding of patients. This study underscores the need for implementing validated questionnaires and observer calibration.

Additional prospective studies could confirm the consistency of these results.

### 5.1. Implications for Practice

From a clinical standpoint, the findings of this study highlight key considerations for optimizing patient management, emphasizing the need for healthcare professionals to integrate a detailed evaluation of possible risk factors associated with tooth wear into routine practice and evaluate the need to or the benefit of proceeding with restorative procedures.

Additionally, this study underscores the role of specific risk factors in influencing the progression of tooth wear, suggesting that targeted interventions in order to manage and control these variables should be prioritized.

This reinforces the need for multidisciplinary collaboration, ensuring that patients receive comprehensive and evidence-based care.

### 5.2. Implications for Research

Future research, ideally with a prospective design, should prioritize subgroup analyses based on the type of tooth wear and the impact of various factors (e.g., presence of bruxism, use of a mouthguard and presence of reflux) on its progression. This approach would enable more precise estimations and facilitate future comparisons. Additionally, incorporating diagnostic and monitoring tools, such as intraoral scanners, is recommended to enhance the accuracy of tooth wear measurements, allowing for better assessment of its rate, progression and associated risk factors.

## Figures and Tables

**Table 1:** Prevalence and distribution of the different types of tooth wear: erosion, attrition, non-carious cervical lesions (NCCL) and their combinations (*) as a function of gender.

	Tooth Wear Type							
	Erosion	Attrition	NCCL	Erosion + Attrition	Erosion + NCCL	Attrition + NCCL	Erosion + Attrition + NCCL	No Wear
Total (*n*= 2266)	62 (2.7)	547 (24.1)	262 (11.6)	54 (2.4)	40 (1.8)	235 (10.4)	42 (1.9)	1026 (45.3)
Female (*n* = 1330)	38 (2.9)	304 (22.9)	159 (12.0)	28 (2.1)	25 (1.8)	120 (9.0)	21 (1.6)	635 (47.7)
Male (*n* = 936)	24 (2.6)	243 (26.0)	103 (11.0)	26 (2.8)	15 (1.6)	115 (12.3)	21 (2.2)	391 (41.8)
*p*-Value	0.674	0.089	0.486	0.473	0.622	**0.012**	0.248	-

(*) Data presented as *n* (% from line total); *p*-values are from Chi-Square test; significant *p*-values (*p* < 0.05) denoted in bold.

**Table 2:** Prevalence and distribution of the different evaluated risk factors (*) as a function of gender.

Risk Factor	Total	Female	Male	*p*-Value
Sjögren’s Syndrome	7 (0.3)	6 (0.5)	1 (0.1)	0.251
Bronchodilator	38 (1.7)	22 (1.7)	16 (1.7)	0.920
Antacids	21 (0.9)	12 (0.9)	9 (1.0)	0.885
Alcohol intake	197 (8.7)	57 (4.3)	140 (15.0)	**<0.001**
Narcotics	68 (3.0)	18 (1.4)	50 (5.3)	**<0.001**
Fruits (2–3 times/day)	38 (1.7)	29 (2.2)	9 (1.0)	**0.026**
Soda (2–3 times/day)	4 (0.2)	1 (0.1)	3 (0.3)	0.313
Brushing (>3 times/day)	98 (4.3)	61 (4.6)	37 (4.0)	0.465
Mouthwash	953 (42.1)	586 (44.1)	367 (39.2)	**0.021**
Listerine^®^	525 (23.2)	303 (22.8)	222 (23.7)	0.603
Mastication difficulties	438 (19.3)	283 (21.3)	155 (16.6)	**0.005**
Being ashamed to smile	326 (14.4)	231 (17.4)	95 (10.1)	**<0.001**
Frequent dry mouth	265 (11.7)	176 (13.2)	89 (9.5)	**0.007**
Decreased DVO	512 (22.6)	304 (22.9)	208 (22.2)	0.722
Total	2266	1330	936	-

(*) Data presented as *n* (% from column total); *p*-values are from Chi-Square/Fisher’s exact test; significant *p*-values (*p* < 0.05) denoted in bold. DVO—Dimension of Vertical Occlusion.

**Table 3:** Results of the multivariate logistic regression analysis (*) for factors associated with tooth wear types (*n* = 2266).

Tooth Wear Type	Risk Factor (s)	OR (95% CI)	*p*-Value
Erosion	None	-	-
Attrition	Age	1.01 (1.00–1.02)	<0.001
NCCL	Age	1.01 (1.00–1.02)	0.004
Erosion + Attrition	Age	1.03 (1.02–1.05)	<0.001
	Antacids	7.07 (1.95–25.62)	0.003
	Mastication difficulties	1.87 (1.03–3.39)	0.039
Erosion + NCCL	Age	1.03 (1.01–1.05)	<0.001
Attrition + NCCL	Age	1.05 (1.04–1.06)	<0.001
	Drugs	2.38 (1.08–5.25)	0.032
	Mouthwash use	1.47 (1.11–1.95)	0.008
	Gender (Female)	0.70 (0.53–0.93)	0.015
Erosion + Attrition + NCCL	Age	1.05 (1.03–1.07)	<0.001
	Decreased DVO	2.16 (1.09–4.30)	0.028

(*) Final reduced logistic regression models, obtained through a stepwise procedure; OR—Odds Ratio; 95% CI—95% Confidence Interval for OR; DVO—Dimension of Vertical Occlusion; NCCL—non-carious cervical lesion.

## Data Availability

The data supporting the reported results are confidential and cannot be shared publicly due to privacy and ethical restrictions. However, interested researchers may request access by contacting the corresponding author via email. Access will be granted at the discretion of the authors and in accordance with applicable regulations.

## Abbreviations

The following abbreviations are used in this manuscript:

TW	Tooth wear
NCCL	Non-carious cervical lesion
DVO	Dimension of Vertical Occlusion
EMDC	Egas Moniz Dental Clinic
GERD	Gastroesophageal reflux disease

## References

[1] Shellis R.P., Addy M. (2014). The Interactions between Attrition, Abrasion and Erosion in Tooth Wear. Monogr. Oral Sci..

[2] Wetselaar P., Lobbezoo F. (2016). The Tooth Wear Evaluation System: A Modular Clinical Guideline for the Diagnosis and Management Planning of Worn Dentitions. J. Oral Rehabil..

[3] Schlueter N., Amaechi B.T., Bartlett D., Buzalaf M.A.R., Carvalho T.S., Ganss C., Hara A.T., Huysmans M.-C.D., Lussi A., Moazzez R. (2020). Terminology of Erosive Tooth Wear: Consensus Report of a Workshop Organized by the ORCA and the Cariology Research Group of the IADR. Caries Res..

[4] Al-Ani Z. (2023). Tooth Wear: Intrinsic and Extrinsic Mechanical Factors. Dent. Update.

[5] Holbrook W.P., Árnadóttir I.B., Kay E.J. (2003). Prevention. Part 3: Prevention of Tooth Wear. Br. Dent. J..

[6] Loomans B., Opdam N., Attin T., Bartlett D., Edelhoff D., Frankenberger R., Benic G., Ramseyer S., Wetselaar P., Sterenborg B. (2017). Severe Tooth Wear: European Consensus Statement on Management Guidelines. J. Adhes. Dent..

[7] Wetselaar P., Wetselaar-Glas M.J.M., Katzer L.D., Ahlers M.O. (2020). Diagnosing Tooth Wear, a New Taxonomy Based on the Revised Version of the Tooth Wear Evaluation System (TWES 2.0). J. Oral Rehabil..

[8] Ferrando Cascales Á., Sauro S., Hirata R., Astudillo-Rubio D., Ferrando Cascales R., Agustín-Panadero R., Delgado-Gaete A. (2023). Total rehabilitation using adhesive dental restorations in patients with severe tooth wear: A 5-year retrospective case series study. J. Clin. Med..

[9] Awad M.A., El Kassas D., Al Harthi L., Abraham S.B., Al-Khalifa K.S., Khalaf M.E., Al Habashneh R., Bartlett D. (2019). Prevalence, Severity and Explanatory Factors of Tooth Wear in Arab Populations. J. Dent..

[10] Van’t Spijker A., Rodriguez J.M., Kreulen C.M., Bronkhorst E.M., Bartlett D.W., Creugers N.H.J. (2009). Prevalence of Tooth Wear in Adults. Int. J. Prosthodont..

[11] Zhang Q., Witter D.J., Bronkhorst E.M., Bartlett D.W., Creugers N.H.J. (2014). Occlusal Tooth Wear in Chinese Adults with Shortened Dental Arches. J. Oral Rehabil..

[12] Bartlett D., O’Toole S. (2021). Tooth Wear: Best Evidence Consensus Statement. J. Prosthodont..

[13] Kreulen C.M., Van’T Spijker A., Rodriguez J.M., Bronkhorst E.M., Creugers N.H.J., Bartlett D.W. (2010). Systematic Review of the Prevalence of Tooth Wear in Children and Adolescents. Caries Res..

[14] Salas M.M.S., Nascimento G.G., Huysmans M.C., Demarco F.F. (2015). Estimated Prevalence of Erosive Tooth Wear in Permanent Teeth of Children and Adolescents: An Epidemiological Systematic Review and Meta-Regression Analysis. J. Dent..

[15] Lourenço M., Azevedo Á., Brandão I., Gomes P.S. (2018). Orofacial Manifestations in Outpatients with Anorexia Nervosa and Bulimia Nervosa Focusing on the Vomiting Behavior. Clin. Oral Investig..

[16] Manarte P., Conceição Manso M., Souza D., Frias-Bulhosa J., Gago S. (2009). Analytic Cross-Sectional Study on Dental Erosion Experience. Med. Oral Patol. Oral Cir. Bucal.

[17] Al-Ani B.Z.N. (2019). The Prevalence and Associated Factors of Tooth Wear Amongst Adults Seeking Treatment in the Faculty of Dentistry, University of Malaya. Master’s Thesis.

[18] Hegde M., Yelapure M., Honap M., Devadiga D. (2018). The prevalence of tooth wear and its associated risk factors in Indian South West coastal population: An epidemiological study. J. Int. Clin. Dent. Res. Organ..

[19] Hemmings K., Truman A., Shah S., Chauhan R. (2018). Tooth wear guidelines for the bsrd part 1: Aetiology, diagnosis and prevention. Dent. Update.

[20] Atalay C., Ozgunaltay G. (2018). Evaluation of tooth wear and associated risk factors: A matched case–Control study. Niger. J. Clin. Pract..

[21] Nadiger R. (2012). Tooth Wear, Etiology, Diagnosis and Its Management in Elderly: A Literature Review. Int. J. Prosthodont. Restor. Dent..

[22] Kontaxopoulou I., Alam S. (2015). Risk Assessment for Tooth Wear. Prim. Dent. J..

[23] Radzki D., Wilhelm-Węglarz M., Pruska K., Kusiak A., Ordyniec-Kwaśnica I. (2022). A Fresh Look at Mouthwashes—What Is Inside and What Is It For?. Int. J. Environ. Res. Public Health.

[24] Shrestha D., Rajbhandari P. (2018). Prevalence and Its Associated Risk Factors in Tooth Wear. J. Nepal Med. Assoc..

[25] Sun K., Wang W., Wang X., Shi X., Si Y., Zheng S. (2017). Tooth wear: A cross-sectional investigation of the prevalence and risk factors in Beijing, China. BDJ Open.

[26] Wei Z., Du Y., Zhang J., Tai B., Du M., Jiang H. (2016). Prevalence and Indicators of Tooth Wear among Chinese Adults. PLoS ONE.

[27] Zhang J., Du Y., Wei Z., Tai B., Jiang H., Du M. (2015). The prevalence and risk indicators of tooth wear in 12- and 15-year-old adolescents in Central China. BMC Oral Health.

[28] Sadaf D., Ahmad Z. (2014). Role of Brushing and Occlusal Forces in Non-Carious Cervical Lesions (NCCL). Int. J. Biomed. Sci..

[29] Bartlett D.W., Lussi A., West N.X., Bouchard P., Sanz M., Bourgeois D. (2013). Prevalence of tooth wear on buccal and lingual surfaces and possible risk factors in young European adults. J. Dent..

[30] Strużycka I., Lussi A., Bogusławska-Kapała A., Rusyan E. (2017). Prevalence of erosive lesions with respect to risk factors in a young adult population in Poland—A cross-sectional study. Clin. Oral Investig..

[31] Ravelli M.N., Schoeller D.A. (2020). Traditional Self-Reported Dietary Instruments Are Prone to Inaccuracies and New Approaches Are Needed. Front. Nutr..

[32] Ackerman B., Siddique J., Stuart E.A. (2019). Transportability of Outcome Measurement Error Correction: From Validation Studies to Intervention Trials. arXiv.

[33] Donaldson M., Goodchild J.H. (2006). Oral health of the methamphetamine abuser. Am. J. Health-Syst. Pharm..

[34] Shetty V., Mooney L.J., Zigler C.M., Belin T.R., Murphy D., Rawson R. (2010). The Relationship Between Methamphetamine Use and Increased Dental Disease. J. Am. Dent. Assoc..

[35] Zwier N., Huysmans M.C.D.N.J.M., Jager D.H.J., Ruben J., Bronkhorst E.M., Truin G.J. (2013). Saliva Parameters and Erosive Wear in Adolescents. Caries Res..

[36] Direção-Geral da Saúde (2022). Estudo de Adesão ao Padrão Alimentar Mediterrânico.

[37] Augimeri G., Caparello G., Caputo I., Reda R., Testarelli L., Bonofiglio D. (2024). Mediterranean Diet: A Potential Player in the Link between Oral Microbiome and Oral Diseases. J. Oral Microbiol..

[38] Louro T., Simões C., Penetra M.J., Carreira L., Castelo P.M., Luis H., Moreira P., Lamy E. (2021). Relationship between Mediterranean Diet Adherence and Saliva Composition. Nutrients.

[39] Wiener R.C., Wu B., Crout R., Wiener M., Plassman B., Kao E., McNeil D. (2010). Hyposalivation and Xerostomia in Dentate Older Adults. J. Am. Dent. Assoc..

[40] Bong S., Lee K., Dominici F. (2024). Differential recall bias in estimating treatment effects in observational studies. Biometrics.

[41] El Aidi H., Bronkhorst E.M., Huysmans M., Truin G.J. (2011). Multifactorial analysis of factors associated with the incidence and progression of erosive tooth wear. Caries Res..

[42] Ganss C., Klimek J., Lussi A. (2006). Accuracy and consistency of the visual diagnosis of exposed dentine on worn occlusal/incisal surfaces. Caries Res..

